# Feeding biomechanics suggests progressive correlation of skull architecture and neck evolution in turtles

**DOI:** 10.1038/s41598-020-62179-5

**Published:** 2020-03-26

**Authors:** Gabriel S. Ferreira, Stephan Lautenschlager, Serjoscha W. Evers, Cathrin Pfaff, Jürgen Kriwet, Irena Raselli, Ingmar Werneburg

**Affiliations:** 1 Faculdade de Filosofia, Ciências e Letras de Ribeirão Preto, Universidade de São Paulo, Av. Bandeirantes 3900, 14040-901 Ribeirão Preto, Brazil; 2 Fachbereich Geowissenschaften der Eberhard Karls Universität Tübingen, Hölderlinstraße 12, 72074 Tübingen, Germany; 3 School of Geography, Earth and Environmental Sciences, University of Birmingham, B15 2TT Birmingham, United Kingdom; 4 Department of Earth Sciences, University of Oxford, South Parks Road, Oxford, OX1 3AN UK; 5 University of Vienna, Department of Palaeontology, Althanstraße 14, 1090 Vienna, Austria; 6 Jurassica Museum, Route de Fontenais 21, 2900 Porrentruy, Switzerland; 7 Department of Geosciences, University of Fribourg, Chemin du musée, 1700 Fribourg, Switzerland; 8 Senckenberg Center for Human Evolution and Palaeoenvironment (HEP) an der Eberhard Karls Universität, Sigwartstraße 10, 72076 Tübingen, Germany

**Keywords:** Palaeontology, Biomechanics, Herpetology

## Abstract

The origin of turtles is one of the most long-lasting debates in evolutionary research. During their evolution, a series of modifications changed their relatively kinetic and anapsid skull into an elongated akinetic structure with a unique pulley system redirecting jaw adductor musculature. These modifications were thought to be strongly correlated to functional adaptations, especially to bite performance. We conducted a series of Finite Element Analyses (FEAs) of several species, including that of the oldest fully shelled, Triassic stem-turtle *Proganochelys*, to evaluate the role of force distribution and to test existing hypotheses on the evolution of turtle skull architecture. We found no support for a relation between the akinetic nature of the skull or the trochlear mechanisms with increased bite forces. Yet, the FEAs show that those modifications changed the skull architecture into an optimized structure, more resistant to higher loads while allowing material reduction on specific regions. We propose that the skull of modern turtles is the result of a complex process of progressive correlation between their heads and highly flexible necks, initiated by the origin of the shell.

## Introduction

**Figure 1:**
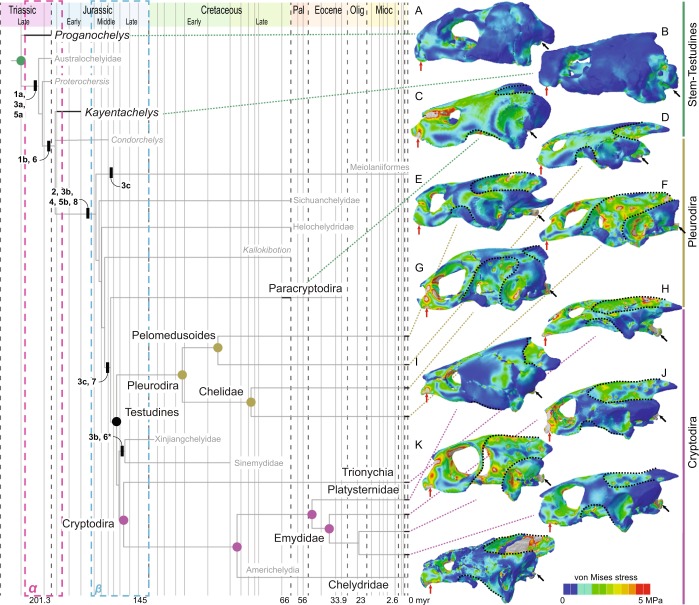
Stress plots resulting from biomechanical analysis of turtles simulated for bilateral biting. (**A–M**), von Mises stress contour plots on a time-calibrated phylogeny (modified from Joyce *et al*.^27^). (**A**), †*Proganochelys quenstedtii*, (**B**), †*Kayentachelys aprix*, (**C**), †*Eubaena cephalica*, (**D**), *Podocnemis expansa*, (**E**), *Pelomedusa subrufa*, (**F**), *Chelodina oblonga*, (**G**), *Emydura subglobosa*, (**H**), *Pelodiscus sinensis*, (**I**), *Platysternon megacephalum*, (**J**), *Graptemys geographica*, (**K**), *Terrapene carolina*, (**L**), *Emys orbicularis*, (**M**), *Chelydra serpentina*. Contour plots are scaled to the same size and 5 MPa peak stress. Dotted curves represent the margins of the emarginations. The purple “α” and the blue “β” rectangles represent the first and second proposed selective regimes described below and on Fig. 5. Caption: 1a, enlarged otic chamber, but shallow tympanic cavity; 1b, enlarged otic chamber and deep tympanic cavity (largest); 2, adductor chamber extending posterior to otic chamber; 3a, basipterygoid process sutured and facing ventrally (low possible kinesis); 3b, basipterygoid process sutured and facing laterally (lower possible kinesis); 3c, basipterygoid process absent (definitive akinesis); 4, secondary lateral braincase wall; 5a, reduced interpterygoid vacuities; 5b, closed interpterygoid vacuities; 6, reduced foramen palatinum posterius; 7, reduced temporal roof by emarginations; 8, trochlear process; *, reversals.

The vertebrate skull is a complex composite of various integrated structures, which are highly integrated to feeding, behavior, and ecology^1,2^. The skull of turtles strongly differs from that of other reptiles^3,4^, which, together with the reorganization of the postcranium by the origin of the shell^5^, has hampered an assessment of their phylogenetic origin^6^. The anapsid morphology (although secondarily acquired^7^) was altered by marginal reductions of dermatocranial bones on the temporal skull region (called emarginations; Supplementary Fig. 9^8,9^), which superficially resemble excavated temporal fenestrae of other amniote groups^4^. The cranial kinesis of early stem-turtles (e.g., †*Proganochelys quenstedtii*^10^) was later lost by a series of modifications (Fig. 1) that stiffened their skulls, similar to mammals and crocodiles^11,12^: (*a*) the fixation of the palatoquadrate to the braincase, by suturing the joint between the parabasisphenoid to the pterygoid found in early turtles (Supplementary Fig. 9f,g)^11,13^; (*b*) fixation of the snout by extensive ossification of the palate, reduction of the foramen palatinum-posterius, and closure of the interpterygoid vacuities (Supplementary Fig. 9f)^12^; and (*c*) development of a secondary lateral braincase wall by a descending process of the parietal (and sometimes also by the epipterygoid) that reaches the palatine and pterygoid ventrally^8^.

Despite gross similarities between the cranial evolution of turtles, mammals, and crocodiles, distinct explanations have been invoked for the evolution of their akinetic skulls: the specific mammalian breathing and chewing mechanisms of neonates^14^, and the outstanding high bite forces of crocodilians^15^ are thought to be the main drivers of cranial evolution in these groups. The reduction of temporal coverage, which is thought to be related to jaw muscle performance in most reptiles^4,9,16^, has been hypothesized to be strongly correlated to neck flexibility and retraction modes in different turtle lineages^17–19^. Werneburg & Maier^12^ presented a hypothesis explaining the reduction of intracranial kinesis in turtles by influences of embryonic neck movements on the fixation of the palatoquadrate to the braincase. This hypothesis suggests that the reorganization of the neck muscles and the development of high flexibility and neck retraction related to the origin of the shell might have had profound influences on the peculiar skull architecture of turtles^17^. Importantly, the rapid diversification of the group since the Middle Jurassic^20^ followed the acquisition of akinetic, emarginated skulls, and longer, more flexible necks^11,18,19^, perhaps representing a case of an adaptive radiation.

Turtles possess a unique jaw closure mechanism, which redirects adductor muscles around the enlarged otic chamber via a trochlear system^11,21^. Horizontal external jaw adductor muscle fibers, which are absent in most amniotes^22–24^, are anteriorly redirected via the trochlea to be vertically inserted onto the lower jaw. This pulley system includes hard and soft tissue components: a transiliens cartilage that slides on a bone surface, facilitated by a synovial capsule or an infold of the mouth cavity^3,22^. Cryptodira and Pleurodira, the extant lineages of crown-group turtles, each exhibiting the trochlea in different positions—the former by a roughening or a process on the otic chamber itself and the latter by a lateral projection of the pterygoid^22^, derived from the external process of the pterygoid (Supplementary Fig. 9). Historically, the position of the trochlea was used to assign fossil turtles to one of these two crown-clades^25^. However, more recent phylogenetic analyses^26,27^ have consistently retrieved a longer stem-lineage to both lineages revealing that the “cryptodiran”-type trochlea appeared along the turtle stem, likely representing also the ancestral pleurodiran condition^28^.

Both the origin of a pulley system and its subsequent modification in pleurodires have been tentatively explained on functional grounds. The origin of the mechanism could have triggered the posterior elongation of muscle fibers along with supraoccipital and squamosal crests as origin sites, resulting in stronger bites^11^. The pterygoid expansion would position the trochlea more anteriorly, allowing the fibers to insert more vertically on the lower jaw, which might result in a more efficient force transfer system^21^. The reinforcement of the skull in turtles has also been suggested to withstand higher bite forces enabling the development of a higher muscle volume^11^. However, none of these hypotheses have been explicitly tested with biomechanical models up to now.

Although mechanical adaptation to functional needs have been considered the main explanation for bone shape, it is clear that phylogenetic, ontogenetic, and architectural constraints bound the actual extent to which skeletal structures can be functionally optimized^29^. In this framework, Finite Element Analysis (FEA) is used to model biomechanical behavior and deformation of interconnected complex structures and to explore the loading history that shaped the morphology of a given structure during its evolutionary history^29–31^. Here, we aimed to assess the functional significance of changes in turtle skull architecture during feeding, using 3D models segmented from micro-CT scans (Supplementary Fig. 9) and a series of FEAs. We tested (*i*) whether taxa with reduced basipterygoid mobility and longer adductor chambers develop higher bite forces^11^, (*ii*) whether the presence of trochlear mechanisms provides biomechanical advantages for the cranial structures^11,21^, and (*iii*) whether an akinetic skull, by redistributing stress, might relief loads on dermatocranial bones on the temporal region enabling their reduction in turtles^17^.

## Results

### General stress distributions

**Figure 2:**
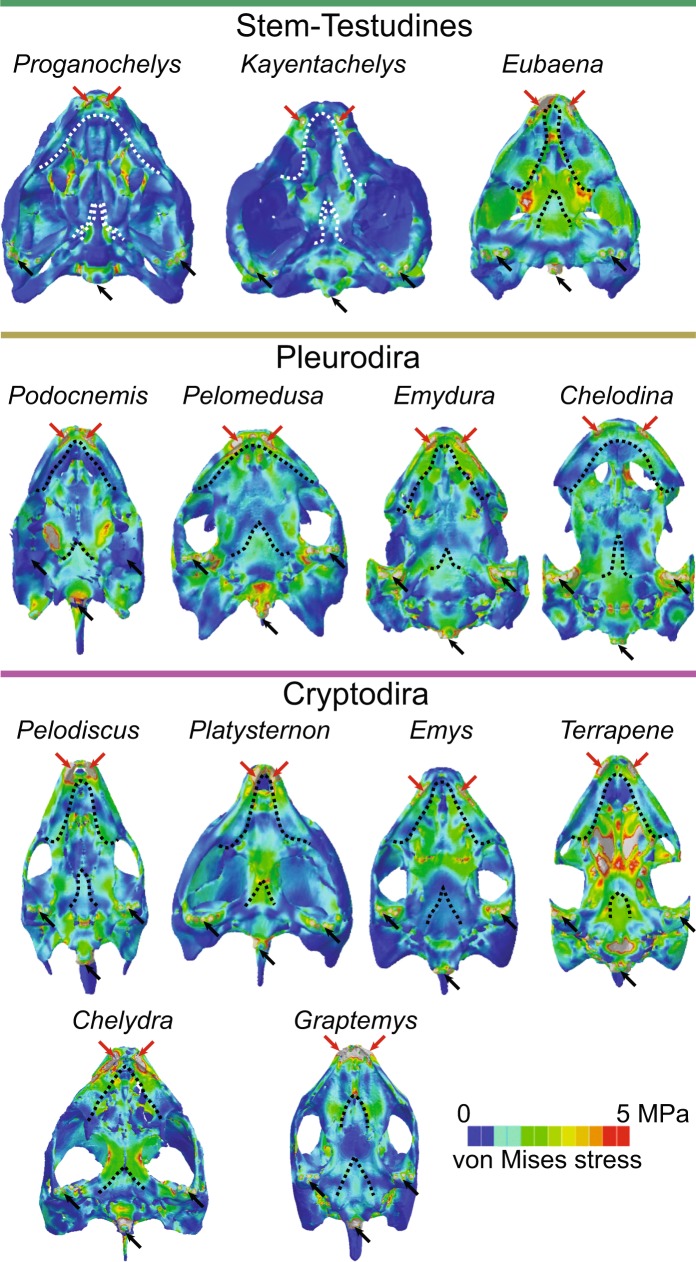
Von Mises stress contour plots of the ventral region of the skull. Contour plots are scaled to the same size and 5 MPa peak stress. Red and black arrows represent the bite and constrained points, respectively. Dotted curves anteriorly and posteriorly, identify the triturating surface and the basipterygoid articulation/suture.

**Figure 3:**
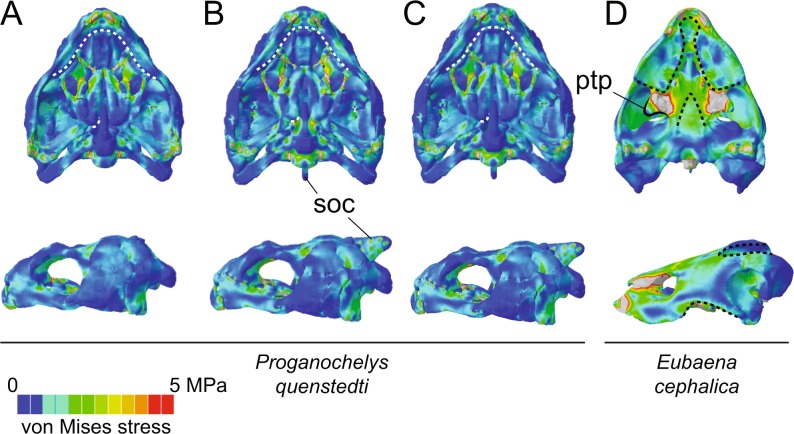
Ventral and left lateral von Mises stress contour plots for hypothetical models of †*Proganochelys quenstedtii* and †*Eubaena cephalica*. †*Proganochelys quenstedtii* with (**A**), basipterygoid suture, (**B**), supraoccipital crest, and (**C**), both modifications; (**D**), †*Eubaena cephalica* with a hypertrophied pterygoid process and both trochlear loads simulated. Contour plots are all scaled to the same size and 5 MPa peak stress. Dotted curves in ventral view, anteriorly and posteriorly, identify the triturating surface and the basipterygoid articulation/suture, and in lateral view the anteroventral and posterodorsal emarginations. Abbreviations: ptp, pterygoid trochlear process; soc, supraoccipital crest.

The von Mises stress contour plots (Figs. 1–3) and average stress measures (Supplementary Fig. 8) show overall lowest stress magnitudes in the early stem turtles †*Proganochelys quenstedtii* and †*Kayentachelys aprix*. Localized stress hotspots aside from the constrained points (i.e., occipital and mandibular condyles and bite points; Figs. 1–2) can be seen anteriorly, inside the orbit (Fig. 1A–B), and on the palate, between the foramen palatinum posterius and the triturating surface (Fig. 2). Additionally, in †*Pr. quenstedtii*, the basipterygoid joint is more loaded than the overall stress pattern (Fig. 2), showing higher degrees of compression (Supplementary Fig. 1).

Extant turtles and †*Eubaena cephalica* are characterized by increased magnitudes of overall stress (Supplementary Fig. 8) in comparison to early stem turtle models (Figs. 1–2). In all of those, regardless of clade affinity (i.e., Pleurodira, Cryptodira, or Paracryptodira; Fig. 1), the dorsal surface of the otic chamber is highly loaded (i.e., high degrees of stress), the basipterygoid suture shows less stress in relation to the rest of the skull, and the bar between the external nares and the orbit is highly loaded (as is also seen in †*Pr. quenstedtii*). In *Pelodiscus sinensis* and *Chelodina reimanni* (Fig. 2) the parabasisphenoid and the basioccipital, which have a more open and interdigitated suture in these taxa, are additional stress hotspots. Taxa with extensive ventrolateral emarginations (i.e., *C. reimanni*, *Terrapene ornata*, and *Emydura subglobosa*) show an increased load laterally on the quadrate, inside the tympanic cavity (Fig. 1). †*Eu. cephalica* and cryptodires (except for *Pelod. sinensis* and *Graptemys geographica*) show higher compression anteriorly on the pterygoid and palatine than pleurodires (Fig. 2). *Chelodina reimanni* and *T. ornata*, both turtles that lost their temporal bridges^9,24^ show higher levels of overall skull stress (Supplementary Fig. 8). The pterygoid trochlear process in pleurodires, as well as the external process of the pterygoid in cryptodires, show very light loads, but in †*Eu. cephalica*, this region is highly stressed (Fig. 2C). Although the dorsal surface of the otic chamber is more loaded than the overall stress distribution, the trochlear process is not particularly stressed in cryptodires and in †*Eu. cephalica*, even when an extra trochlea, on the pterygoid process, is explicitly simulated in the latter (Supplementary Figs. 2–7).

### Simulations in *†Proganochelys* and *†Eubaena*

To test the effects of observed evolutionary modifications on the turtle skull, we digitally created hypothetical models of †*Proganochelys quenstedtii*, simulating (*i*) a fixed (=akinetic) basipterygoid joint, and (*ii*) a supraoccipital crest, simulating the posteriorly expanded origin sites of external jaw adductors found in later turtles. Simulations have little effect on the stress distribution in relation to its original model’s contour plots (Figs. 2–3, Supplementary Fig. 1). Closing the basipterygoid joint results in a slight increase in overall stress, especially on the skull roof, whereas most significant change is seen on the parabasisphenoid, which experiences much less stress than in the original model. Additionally, the area around the foramen palatinum posterius shows slightly decreased loads in this hypothetical model (Fig. 3A).

Similarly, modeling a supraoccipital crest (Fig. 3B, Supplementary Fig. 1), yielded only minor differences: the skull roof is somewhat more loaded, with more concentrated areas of stress on the sutures of the frontal to the parietal and postorbital, whereas the ‘cheek’ area (jugal/quadratojugal) is less stressed. More importantly, the basipterygoid joint and the parabasisphenoid as a whole are more stressed in comparison to the original model. A third †*Pr. quenstedtii* model combining both modifications (sutured basipterygoid joint and extended supraoccipital crest) yielded a stress release on both the parabasisphenoid and on the cheek region (Fig. 3C).

We also modeled an additional pterygoid trochlear process and trochlear load on the †*Eubaena cephalica* model to simulate an intermediate morphotype with two trochleae, as proposed by Joyce^21^. In contrast to the †*Pr. quenstedtii* models, the hypothetical †*Eu. cephalica* (Fig. 3D, Supplementary Fig. 2) shows significant effects. The external process of the pterygoid shows a much higher load when this trochlea is simulated (Fig. 3D), associated also with a change from compression to tension loads (Supplementary Fig. 2). The cheek region is under less stress (Fig. 3D) in comparison to the original model (Fig. 1C), but the border of the anteroventral emargination is slightly more compressed. It is noteworthy that the basipterygoid joint shows a relief in compression when a pterygoid trochlea is present (Supplementary Fig. 2).

### Bite forces and efficiency estimates

**Figure 4:**
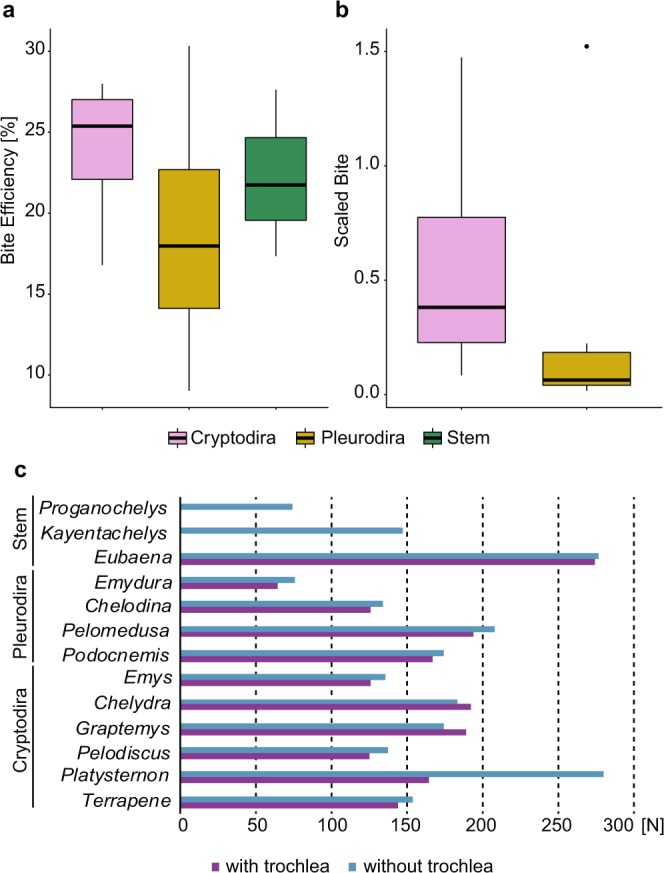
Bite force and efficiency in turtles. (**a–b**), Boxplots of bite efficiency based on (**a**), FEA estimates and (**b**), *in vivo* measurements (scaled bite force calculated by dividing measured bite force^33^ by carapace size). (**c**), Bite force estimates based on FEA for sampled taxa.

Estimates for models simulating trochlear loads in cryptodires and pleurodires show only slight decreases in bite force (1–15%) in relation to the standard models (i.e., models without the trochlear mechanism explicitly modeled; see Methods for full explanation), except for *Platysternon megacephalum*, in which there was a 44% decrease in the estimated bite force (Fig. 4c). Yet, the decrease in bite efficiency was higher in the models with a trochlea: 12–25%, and a 46% decrease for *Pl. megacephalum* (Supplementary Table 1). Cryptodires and pleurodires do not clearly differ (Supplementary Table 2) in their range of bite efficiency (i.e., bite force divided by muscle force) (Fig. 4a), as within-group variation is higher than among-group variation (especially for pleurodires). *Chelodina reimanni* and *Emydura subglobosa* are the least bite efficient models, whereas *Graptemys geographica* and *Podocnemis unifilis* are the most bite efficient models. The bite efficiency (as well as the bite forces) of the stem turtles fell into the spectrum of the analyzed extant taxa (Fig. 4a, Supplementary Table 2).

## Discussion

### Bite force does not seem to increase during turtle evolution

It has been suggested that the origin of the trochlear mechanism in turtles provided a biomechanical advantage enabling more efficient bite performances^11,21^, possibly triggering the diversification of turtles during the Middle Jurassic^20,26,27^ (Fig. 1). Evolutionary innovations prior to the evolution of trochleae included the fixation of the braincase, palatoquadrate, and snout, and the development of an extensive secondary lateral wall of the braincase^11,12^. Trochleae then evolved in crownward parts of the turtle stem lineage, in the clade including Meiolaniformes and Testudines^27^ (Fig. 1). Modifications of the jaw adductor system would represent adaptive innovations related to increasing muscle power and bite forces. This hypothesis predicts that early stem turtles lacking these modifications, such as †*Kayentachelys aprix*^32^, were less powerful biters than modern turtles^11^.

The evolutionary trend towards the akinetic skull of Testudines began early in turtle evolution^11,13^. †*Proganochelys quenstedtii* had a relatively kinetic skull, with open basipterygoid joint, but †*Kay. aprix* (Figs. 1–2) shows decreased basipterygoid movement along a sutured (but unfused) basipterygoid articulation^32^. Our FE models show that palatal and basipterygoid areas were under high stress in †*Pr. quenstedtii*, but in †*Kay. aprix* and other testudinates they became less stressed at the same time that more extensive sutures developed (Fig. 2). However, unlike in the case of crocodilian evolution^15^, skull stiffening in turtles is associated only with a moderate increase in bite forces. Similarly, the posterior elongation of the adductor chamber that occurred in turtles more crownward than †*Kay. aprix* (Fig. 1), does not seem to have considerably increased bite force or efficiency, in opposition to previous hypotheses^11^: estimates based on our models show †*Kay. aprix* within the range of Testudines (Fig. 4), and simulating a supraoccipital crest on a †*Pr. quenstedtii* model (Supplementary Fig. 1) does not result in higher bite force values (Supplementary Table 1). The distribution of bite force and bite efficiency shows no clear differences between stem and crown turtles that lack and possess temporal crests, respectively (Fig. 4). This might seem counterintuitive since the posterior elongation of the adductor musculature seems to result in longer and hence stronger fibers. However, the concomitant enlargement of the otic chamber in turtles^8,21^ decreased the available volume inside the adductor chamber, so posterior elongation may have emerged as a compensation for that. Alternatively, the posterior elongation of the adductor chamber could be related to changes in speed, not necessarily to force only, as a trade-off related to fiber length exists between force and speed^33^. This offers an explanation for adductor chamber elongation that does not require stronger bite forces, and could explain the observations in our FE models (Fig. 4) and in experimental studies^33^.

Finally, explicitly simulating loads on the trochlear process (in the otic chamber in cryptodires and in the pterygoid in pleurodires) in our models actually decreases the estimated bite force (Fig. 4c), which can be explained by the physical attributes of the trochlea, according to which part of the muscle force will be lost during attrition of the transiliens cartilage^22^ to the surface of the bone process. Empirical studies show that turtles possess similar bite forces to other reptiles^33^ (excluding crocodiles). Thus, there is no empirical support to assume that the origin of the temporal crests and the trochlear system along the stem to modern turtles resulted in more powerful jaws in comparison to their non-turtle ancestors. However, the changed line of action of the external jaw adductor muscles^3,22^ due to the presence of a trochlear mechanism may likely have a more substantial effect on the lever mechanics of the mandible. This might be affected by the height and by the anteroposterior position of the trochlea (i.e., on the otic chamber or on the pterygoid), which might change the position of the insertion site and, hence, the lever on the lower jaw. While this increased efficiency is focused in the mandible, the skull structure might have experienced no or little negative effects despite of the increased loads acting on it. Hence, our bite force estimates based only on skull models reveal only one aspect of the effects of distinct positions of the trochlea on the resulting bite efficiency. Future studies estimating bite forces from lower jaws would allow a further assessment and could complement our results. Here, we focused on the impact of the trochlea on the biomechanical behavior of the skull, with which it is more closely associated than with the mandible.

### Neck flexibility triggers a series of skull modifications

A common line of reasoning proposes that the posterior elongation of the adductor chamber was related to a restricted space inside the adductor chamber, either by the anapsid condition of the skull^21^ or to the hypertrophy of the otic region^25^. This, though, raises the question of why turtles developed posteriorly^3^ rather than dorsally expanded skulls, a much more common way of fiber elongation in reptiles^4^. Considering that extant macrocephalic turtles with high skulls, e.g., *Platysternon megacephalum* (Fig. 1I) and *Chelonia mydas*^34^, are incapable of fully retracting their head and neck inside their shells^12,17^, neck retraction likely represented a functional constraint during turtle evolution. We hypothesize that the origin of posterior crests was also related to the necessity to (at least) maintain ancestral bite performances in a system that accommodates neck retraction. In other words, high, dorsoventrally expanded skulls would be negatively selected in a case in which hiding the head and neck is a protective advantage^33^. In this hypothesis, developing a trochlear system was the only solution to accomplish the posterior elongation, given the presence of an enlarged otic chamber.

**Figure 5:**
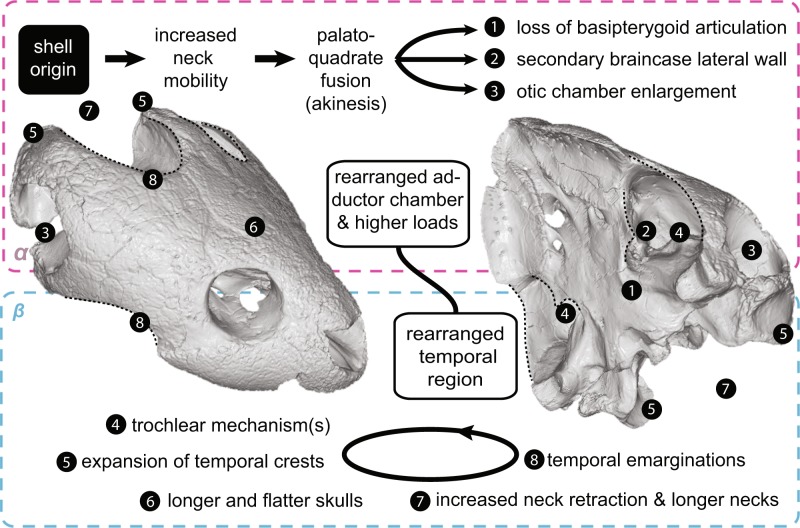
Hypothesis of progressive correlation between neck and head during turtle evolution. The origin of the turtle shell initiates the first selective regime (*α*) in which muscle rearrangements enable more flexible necks that overcome the lack of mobility of the shelled body, but also exert distinct compression and tension loads on the skull. This is compensated by an increasing stiffness (through palatoquadrate fusion, closure of basipterygoid region (1), secondary braincase lateral wall (2) formation). The new, stiffened skull architecture can withstand higher loads at the same time allowing material reduction in the temporal region, opening the path to a second selective regime (*β*). Longer and flatter skulls (6) evolve, together with posterior expansion of temporal crests (5) and the enlarged otic chamber (3), both features reducing available volume inside the adductor chamber and triggering the appearance of a trochlear mechanism (4). Broader insertion sites for neck musculature offered by expanded temporal crests (5) support a new round of neck modifications, resulting in longer necks and modern types of neck retraction (7). Temporal emarginations (8) evolve in response to new forces generated by more powerful neck musculature. Model of †*Eubaena cephalica* as in Supplementary Fig. 9.

Building on previous hypotheses^12,17–19^, we suggest a more detailed scenario for the evolution of the highly modified turtle skull. We propose a progressive correlation between skull architecture and neck evolution (Fig. 5), that incorporates two phases of distinct selective regimes. The first, related to increased neck mobility and skull stiffening, occurring early in the evolution of turtles (*α* in Figs. 1, 5). The second, related to the posterior elongation of the adductor chamber, the pulley system, and longer necks, occurred closer to the radiation of crown Testudines (*β* in Figs. 1, 5).

*(α*) Our results show that the fixation of the snout and the basipterygoid joint, as well as the development of a secondary lateral wall of the braincase (1 & 2 in Fig. 5) during the first phase of our scenario stabilized those regions during feeding movements, which were more fragile (Fig. 2), even though the skull as a whole was more heavily built and stress resistant (Supplementary Fig. 8). Both areas were under high stress in †*Proganochelys quenstedtii* and became alleviated in †*Kayentachelys aprix* (Fig. 2). The †*Pr. quenstedti* hypothetical model with a fixed basipterygoid joint shows reduced stress around this contact supporting our interpretation (Fig. 3C). However, simply stiffening the skull in turtles does not seem to result in higher bite forces since our estimates do not show higher forces or efficiencies in modern turtles in comparison to earlier testudinates (Fig. 4). As such, our biomechanical analyses do not support an increase in bite force as the main factor related to the evolution of akinetic skulls in turtles, in opposition to the proposed case for crocodiles or temnospondyls^15,35^. Evidence has been presented elsewhere for an extensive influence of increased neck mobility—and consequent muscle reorganization—on the fixation of the palatoquadrate to the braincase^12^, and we here propose that this influence might have triggered the path to complete akinetic skulls in turtles (Fig. 5) very early in their evolution (†*Kay. aprix* node in Fig. 1B). Here, the elongation of the adductor chamber might have occurred in response to a decrease in volume due to the enlarged otic chamber (3 in Fig. 5). The first steps of otic region enlargement can be seen as early as in †*Kay. aprix*^32^, which also shows an incipient elongation of the temporal region^21^. Although the secondary enlargement of the otic chamber in crown turtles has been associated with invasion of aquatic environments of Testudines^36^ (but see^37^), its initial enlargement might have been related to the fusion of the quadrate medially to the braincase.

*(β*) The fixation of the palatoquadrate to the braincase resulted in higher overall stress values on the turtle skull (Figs. 1–2), possibly because of the loss of elasticity that could buffer the loads in the whole structure^16^. At the same time, the stiffening created an optimized solution in the sense of higher maximal load resistance (Supplementary Fig. 8) paralleled by a reduction of bone material in particular regions in the skull (Fig. 1). Also, a more resistant skull enabled the quadrate to withstand the compression forces exerted by a trochlear mechanism on its surface (4 in Fig. 5)^11,21^. The trochlear mechanism evolved by bending the adductor musculature around the otic surface with the bone process enabling a synovial articulation with the jaw adductor tendon^38^.

The presence of a shell represented a constraint on skull height due to the need for tucking the head and neck for protection^18,19,33^. Posteriorly expanding the adductor chamber (5 in Fig. 5) was the only way to maintain muscle power by compensating decreased volume by elongated muscle fibers. The trochlear mechanism also changes the vector of the muscle force from this anteroposterior to a more vertical direction. As such, the trochlea enabled the evolution of a posteriorly elongated adductor chamber (5 in Fig. 5) and triggered a shift of the bite efficiency to the mandible without negatively impacting the skull. The crest expansion opened another evolutionary path, allowing skulls to become relatively flatter (6 in Fig. 5). Flatter skulls enhanced skull retraction efficiency (7 in Fig. 5), and the expanded temporal crests provided broader attachment sites for neck musculature as well. By forming emarginations (8 in Fig. 5) in the less loaded temporal region, these attachment sites further increased, enabling even longer and stronger necks. Although progressive correlation is key in our scenario, these factors reciprocally influenced each other in turtle evolution.

### Reduction of the temporal skull coverage

Whereas the first selective regime (*α*) resulted in a rearranged adductor chamber, the second (*β*) resulted in a rearranged temporal region. The lateral and dorsal walls of the adductor chamber in early testudinates exclusively provided the origin sites for the external jaw adductors^3^. The low stress distribution in this region (Fig. 1), however, suggests that it did not represent an important structural component for jaw musculature attachment and could be reduced later in evolution^24^. With the potential to evolve longer and flatter skulls created by skull stiffening (*1, 2, 3, 6* in Fig. 5)—which can be observed in the Jurassic xinjianchelyids^13^ (Fig. 1)—the muscle origin sites are shifted away from the adductor chamber roof to the supraoccipital and squamosal crests^3,23,24^. This and the optimization for minimizing material (described above) enabled the development of marginal reductions of dermatocranial bones on the temporal region (8 in Fig. 5)^3,11,24^, which, in turn, also produced stronger attachment sites for more complex neck muscles (7 in Fig. 5)^17^.

The stabilization of the quadrate was originally secured by the temporal coverage, but the fixation to the braincase released it from this function^9,12^. This event in itself did not diminish, but actually increased stress on specific areas of the temporal roof, especially around the contacts of the jugal, quadratojugal, and squamosal (Supplementary Fig. 1), a pattern also found in extant taxa with extensive temporal coverages, e.g., *Podocnemis unifilis* and *Platysternon megacephalum* (Fig. 1D,I). In most turtles, those areas correspond to the temporal bridge that is almost always maintained between the anterolateral and posterodorsal emarginations^3,24^, suggesting they are important structural components of the turtle skull. The few taxa lacking those bridges, e.g. *Chelodina reimanni* and *Terrapene carolina*, are the ones showing the highest degrees of total stress (Figs. 1–2, Supplementary Fig. 8), supporting this hypothesis. Complex neck movements and retraction modes benefit from more extensive emarginations and broader bridges, which serve as strong and stable neck muscle attachment sites. Thus, only a certain portion of the temporal coverage can be reduced. The increased ossification and reduction in regions that were ancestrally submitted to high or low loads, respectively, illustrate the powerful tool FEA can be for detecting morphological evolutionary patterns in complex structures.

### The pterygoid trochlea in pleurodires

The muscle^3,23,24^ and skull^11–13^ reorganization related to the pterygoid trochlea in pleurodires do not seem to result in more efficient estimated (Fig. 4a) nor empirically measured bites (Fig. 4b)^33^ (contra^21^). Also, cranial competence (i.e., the capacity to sustain stress) is not compromised by the presence of the pterygoid trochlea and other morphological changes in the group (Supplementary Fig. 8). Hence, from the available data we can conclude that the presence of a trochlea on the pterygoid has no substantial effect on the bite force. It is possible, though, that an increase in efficiency can be achieved by the lower jaws (due to changing the position and, to a lesser degree, the angle of attack of the jaw musculature), which are the main functional device for biting. Thus, it can be assumed that the hypothesized increase in bite force is solely achieved by redirecting the muscle insertion angle on the lower jaws^21^; a hypothesis that will be tested by further FEAs based on lower jaw models. The pterygoid trochlea, however, does seem to invoke less stress onto the basicranium and cheek region on the skull (Figs. 1–2). This is supported by our simulation of a ‘pleurodiran’-like trochlea on †*Eu. cephalica* (Fig. 3D), which reduced load in these regions. Even though †*Eu. cephalica* is certainly not an “intermediate” between cryptodiran- and pleurodiran-like morphotypes, it was the best available taxon to model this particular condition, since, as a paracryptodire, it does not represent either the cryptodiran nor the pleurodiran lineages. Of course, simulating this double-trochlea condition in an early pleurodire would be the best option, however, no complete skull of the Panpleurodira platychelyids are currently available^39^ and the oldest known taxa, e.g., the Early Cretaceous †*Atolchelys lepida*, are already well-nested within a lineage with well-developed pleurodiran traits^40^.

In cryptodires and some late stem-turtles, e.g., †*Meiolania platyceps* and †*Kallokibotion bajazidi* (Fig. 1), the pterygoid is posteriorly expanded and closes the cranio-quadrate space^21^. In pleurodires, neck forces during embryonic development reorient the palatoquadrate cartilage and prevent the pterygoid from extending so far posteriorly. The cranio-quadrate space is then closed by the formation of appositional bone, a developmental process called the “Eßwein fixation”^12^. This specific fixation and the resulting skull architecture seem to be related to a distinct stress distribution on the external process of the pterygoid: in cryptodires and †*Eu. cephalica*, the pterygoid process is more loaded than in pleurodires (Fig. 2), even when a pterygoid trochlea is explicitly simulated (Supplementary Fig. 2). The new fixation might have also triggered the external process to expand and assume the function of the trochlea in Pleurodira, which decreased stress on the parabasisphenoid and basioccipital. We hypothesize that even lower loads on the basicranium of pleurodires during biting enabled the expansion of neck muscles onto its ventral surface^12,17,23^ and reduction of the cheek bones^17^ in this group. This association will be further explored by future FEAs on additional skull models including neck musculature.

## Conclusions

Our Finite Element Analyses of turtle skulls do not support the traditional hypotheses that cranial akinesis evolved to accommodate higher bite forces or that the origin of the pleurodiran trochlear system is related to more efficient bites. However, cranial modifications during early turtle evolution resulted in a skull architecture that redistributes stress and allows to resist higher loads while simultaneously allowing for temporal emarginations. The stiffened skull withstands higher loads while also accommodating bone reduction, freeing the potential to acquire more diverse architectures, including longer and flatter heads. These skull modifications do not significantly increase bite forces, but help to maintain them by compensating adductor chamber volume reduction induced by enlarged otic chambers. The evolution of the turtle skull and jaw adductor system is additionally constrained by the retraction of the skull underneath the shell. We integrate our novel observations with the available existing paleontological and anatomical evidence to a scenario of associations between skull architecture and neck evolution: the origin of the turtle shell is related to the evolution of increased neck mobility that compensates the stiffened body while neck retraction offers protection. Associated topological changes of neck muscles are buffered by the fusion of the palatoquadrate to the braincase and consequent closure of the basipterygoid articulation, which was particularly under high stress during bite movements in early testudinates. The modified skull architecture, including skull stiffening, posterior elongation of the adductor chamber, trochlear mechanisms, and the possibility of temporal bone reductions, enabled further modifications of the neck, with musculature attaching to stronger temporal bridges closer to the origin of Testudines. Finally, the potential for the evolution of more disparate skull architectures and longer and more flexible necks opened new adaptive paths for the great diversification of turtles during and after the Jurassic.

## Methods

### Specimens & skull models

Digital models of ten extant and three extinct taxa were segmented from micro-computed tomography (μCT) data (Table S2) using Avizo software (Version 7.0.0, Visualization Science Group). In order to represent more accurately the inferred life morphology, the models of †*Proganochelys quenstedtii* and †*Kayentachelys aprix* required moderate reconstruction. We digitally removed breaks and cracks, corrected distortions and deformations, and missing elements were reflected, following the protocol outlined by Lautenschlager^41^. In addition to the thirteen standard models described above, we also created four additional hypothetical models: (1) †*Pr. quenstedtii* with a palatoquadrate joint, (2) †*Pr. quenstedtii* with a supraoccipital crest, (3) †*Pr. quenstedtii* with a palatoquadrate joint and a supraoccipital crest, and (4) †*Eubaena cephalica* with a hypertrophied pterygoid external process and both trochlea (on the otic chamber and on the pterygoid process) explicitly modeled (Supplementary Figs. 1, 2). Even though there is a considerable temporal gap between †*Pr. quenstedti*, †*K. aprix*, and †*Eu. cephalica*, the morphology in these three taxa exemplify the stepwise acquisition of architectural modifications we were interested in. In order to test the influence of sutures on FE models, we generated two additional models (one †*Pr. quenstedti* and one *Podocnemis unifilis*), in which we segmented the intersutural spaces as separate labels on Avizo. Surface (.stl) files for the final models were generated in Avizo and can be found in the Supplementary Files.

### Mesh models and FEA

The surface models were imported into Hypermesh (Version 11; Altair Engineering) to create solid mesh FE models consisting of ~2,000,000 four-noded tetrahedral elements (tet4) and to set boundary conditions. Material properties were assigned for the cranial bones (and fibrous connective tissue for the additional †*Proganochelys quenstedti* and one *Podocnemis unifilis* with sutures). We used extant analogs for alligator bone (E = 20.49 GPa, υ = 0.40)^42^ and connective tissue (E = 0.09 GPa, υ = 0.30)^43^ with both materials treated as isotropic and homogeneous.

Origin sites and main fiber course for the eight jaw adductor muscles (nine for *Pelodiscus sinensis*, including the m. zygomaticomandibularis^3,23^), obtained from the literature^24^ and personal observations (IW & GSF), were used to assign force vectors to the FE models. To calculate contraction force values for each muscle in our models, we used muscle cross-sectional area based on contrast-enhanced (with PTA, phosphotungstic acid) μCT-scan data of the same extant taxa (except *Graptemys geographica, Pelomedusa subrufa*, *Podocnemis unifilis*, and *Terrapene ornata*, for which we used closely related taxa; Table S3). The models were constrained from rigid body motion in all directions (X, Y, Z) at the occipital (five nodes) and mandibular condyles (four nodes on each side), reflecting attachment to the vertebral column and the lower jaw. To simulate bilateral biting at different analogous positions, additional constraints (one node on each side) were applied to the maxilla, at the tip of the snout near the suture to the premaxilla. An additional set of models was created simulating the effect of the trochlear mechanism on the underlying bone during attrition of the transiliens cartilage. For this purpose, the vectors of the jaw muscles on both sides of the trochlea (i.e., cranial origin site to trochlea and trochlea to mandibular insertion site) were obtained for each taxon. Load force exerted by the trochlea on the bone process was calculated as the vector perpendicular to the resultant vector of the jaw muscles and the trochlea. Explicitly simulating the trochleae in both pleurodires and cryptodires did not change the overall pattern of stress distribution. Similarly, modelling the intracranial bone sutures with different material properties yielded similar results as models with just bone materials. For those reasons, the discussion focuses on the results of the simpler models (called ‘standard models’), i.e. models without simulated trochleae in those groups and without modelled sutures.

For analysis and postprocessing, all FE models were imported into Abaqus (Version 6.10; Simulia) and rescaled to the same size in order to remove the effects of size on bite force estimates and efficiency. Biomechanical performance for each model was assessed via von Mises and tensile/compressive stress contour plot outputs, reaction forces (= bite forces) at the bite points and per-element average stress values.

## Supplementary information

Supplementary information.
