# Supplementary material for: Feeding biomechanics suggests progressive correlation of skull architecture and neck evolution in turtles
Source: Sci Rep. 2020 Mar 26;10:5505. doi: 10.1038/s41598-020-62179-5 (PMC7099039; doi:10.1038/s41598-020-62179-5)
Supplement: Supplementary file 1 — Supplementary information. [file 41598_2020_62179_MOESM1_ESM.pdf]

Supplementary Information

Feeding biomechanics suggests progressive correlation of skull architecture and neck evolution in turtles

Gabriel S. Ferreira[1,2*], Stephan Lautenschlager[3], Serjoscha W. Evers[4,5], Cathrin Pfaff[6], Jürgen Kriwet[6], Irena Raselli[5,7], Ingmar Werneburg[2,8*]

[1]Faculdade de Filosofia, Ciências e Letras de Ribeirão Preto, Universidade de São Paulo, Av. Bandeirantes 3900, 14040-901 Ribeirão Preto, Brazil

[2]Fachbereich Geowissenschaften der Eberhard-Karls-Universität Tübingen, Hölderlinstraße 12, 72074 Tübingen, Germany

[3]School of Geography, Earth and Environmental Sciences, University of Birmingham, B15 2TT Birmingham, United Kingdom

[4]Department of Earth Sciences, University of Oxford, South Parks Road, Oxford, OX1 3AN, UK

[5]Department of Geosciences, University of Fribourg, Chemin du musée, 1700 Fribourg, Switzerland

[6]University of Vienna, Department of Palaeontology, Althanstraße 14, 1090 Vienna, Austria

[7]Jurassica Museum, Route de Fontenais 21, 2900 Porrentruy, Switzerland

[8]Senckenberg Center for Human Evolution and Palaeoenvironment (HEP) an der Eberhard Karls Universität, Sigwartstraße 10, 72076 Tübingen, Germany

[*]Corresponding authors: Gabriel S. Ferreira, gsferreirabio@gmail.com, Ingmar Werneburg, ingmar.werneburg@senckenberg.de

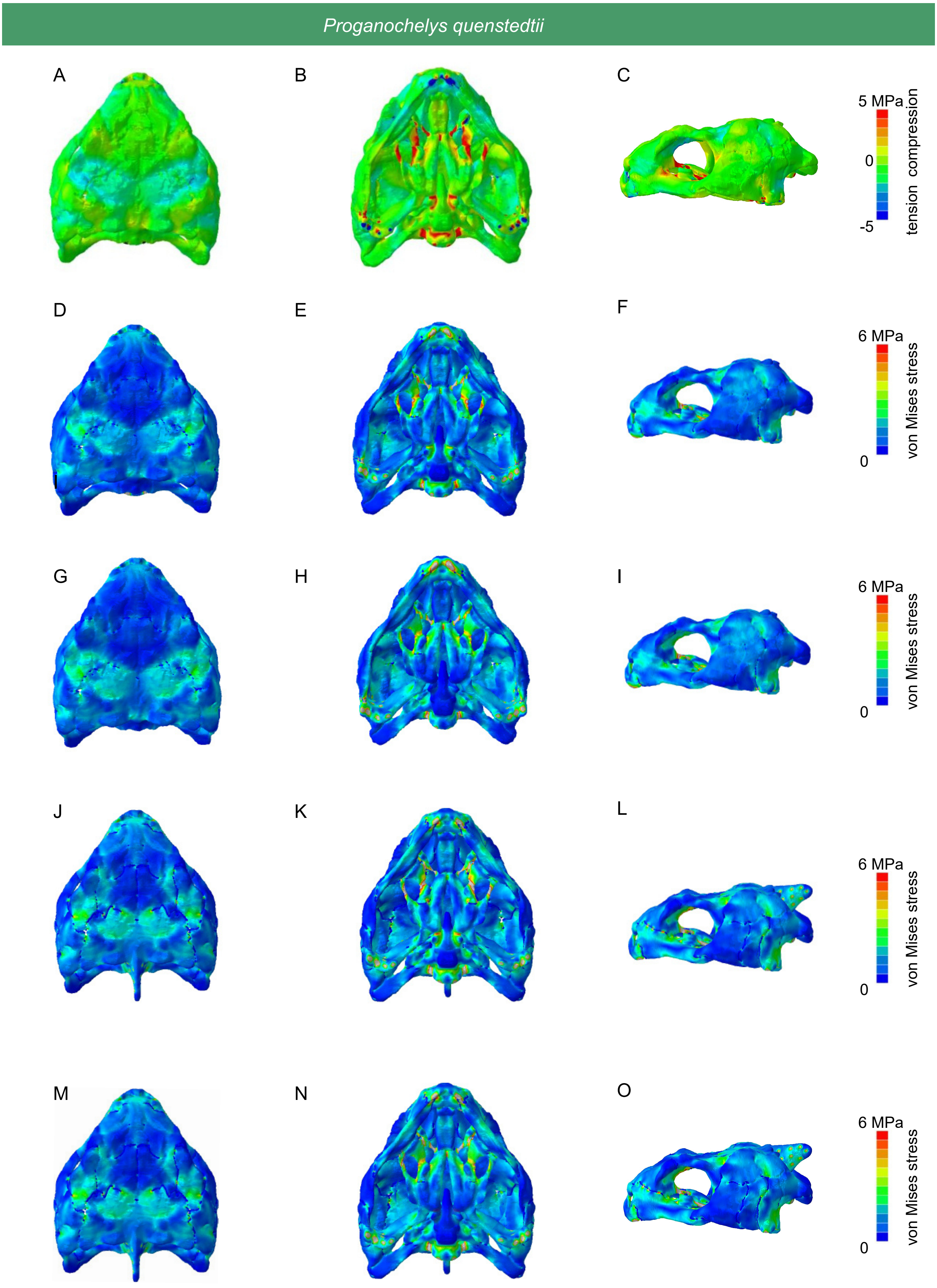

Supplementary Figure 1. Plots of *Proganochelys quenstedtii* in (first column) dorsal, (second) ventral and (third column) left lateral views. **a–c**, tension/compression and **d–o**, von Mises stress contour plots for the original model. **a–f**, original *P. quenstedtii* model, **g–o**, hypothetical models with **g–I**, sutured basipterygoid joint, **j–l**, supraoccipital crest, and **m–o**, sutured basipterygoid joint and supraoccipital crest.

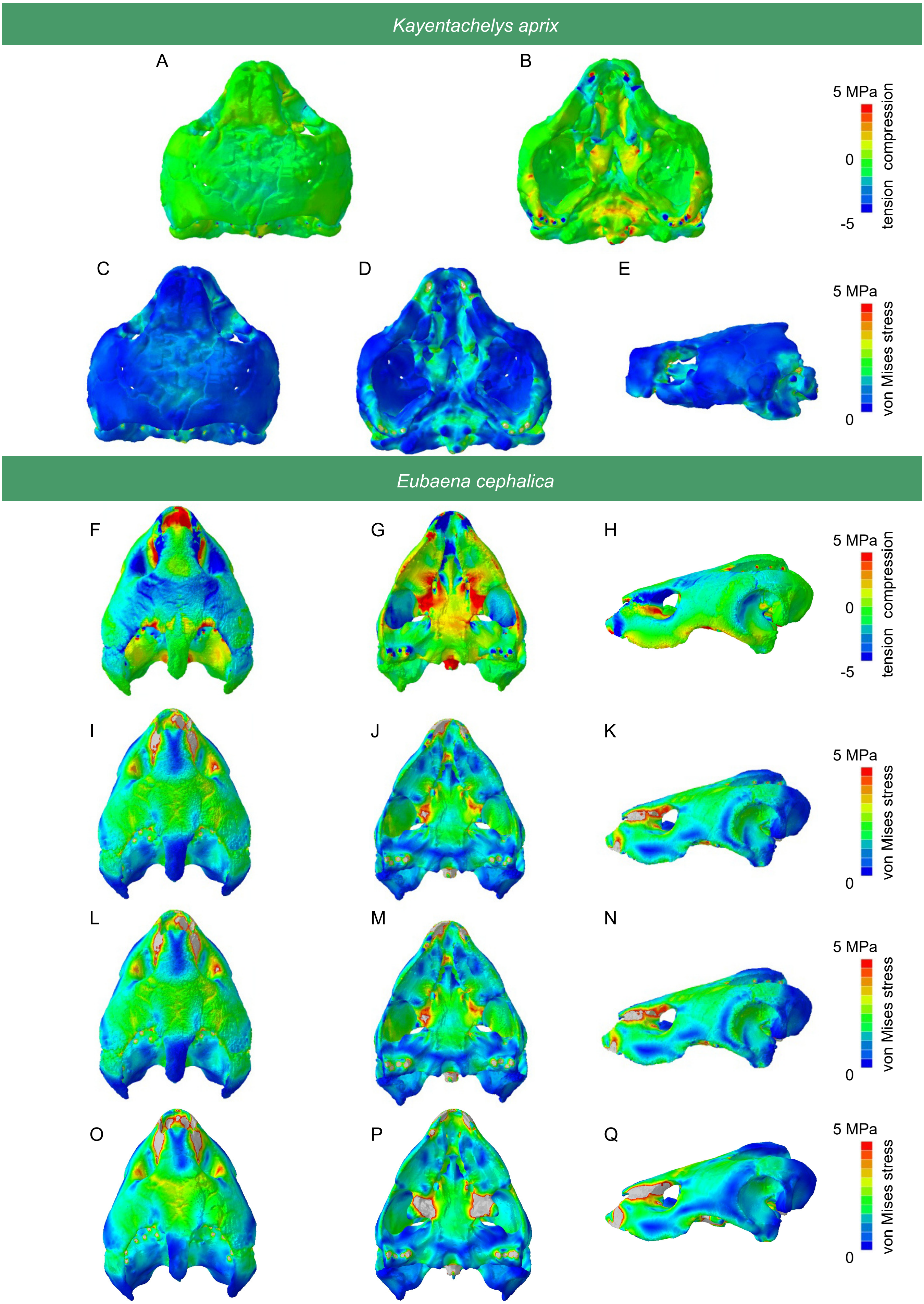

Supplementary Figure 2. a–e, *Kayentachelys aprix* and **f–q**, *Eubaena cephalica* FE models. **a–b**, **f–h**, tension/compression and **c–e**, **i–q**, von Mises stress contour plots in (from left to right) dorsal, ventral and left lateral views. **a–n**, original models and **o–q**, hypothetical *Eubaena cephalica* model with a hypertrophied pterygoid external process and a second trochlear mechanism simulated on it. **l–n**, original model with a simulated otic trochlea.

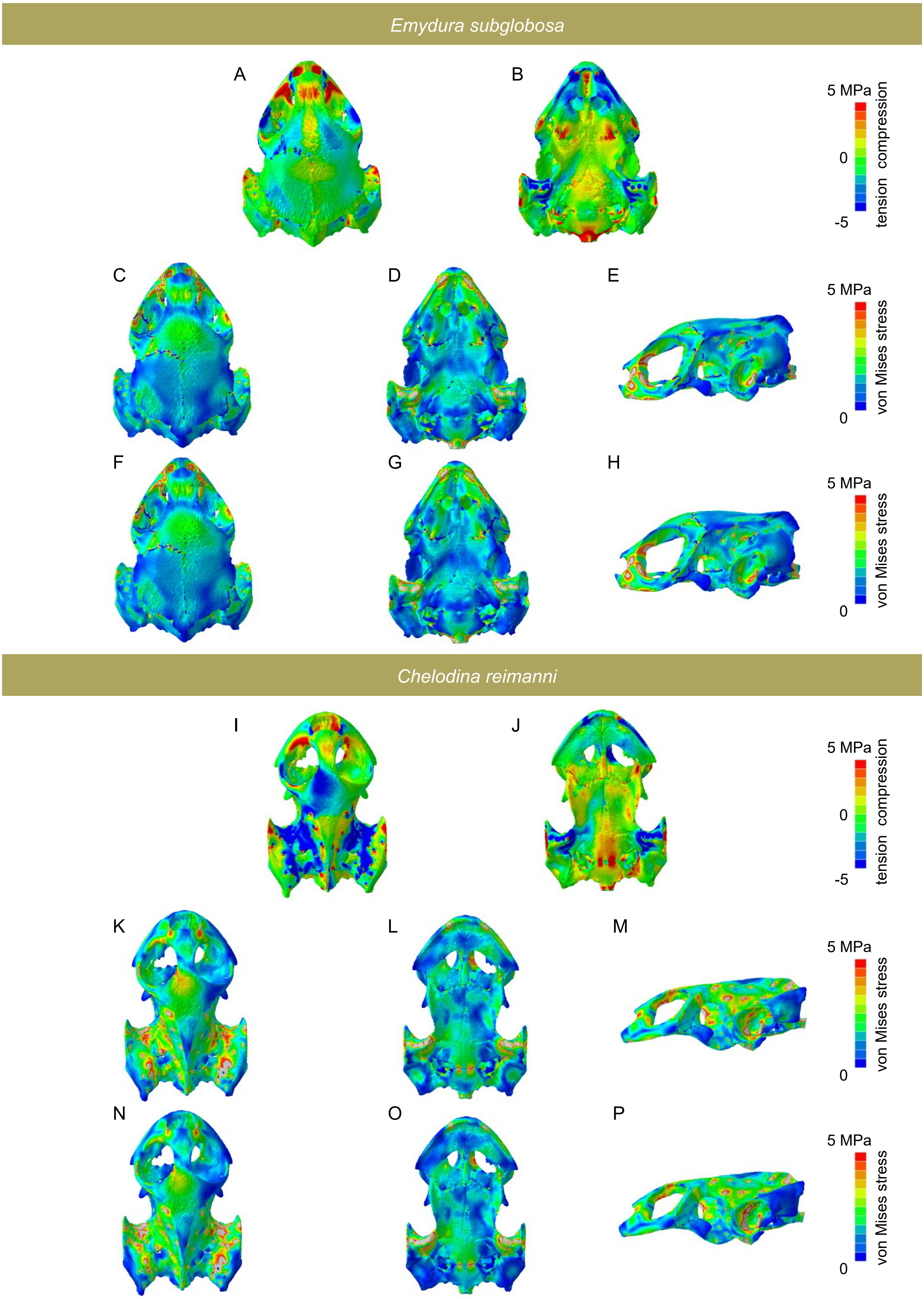

Supplementary Figure 3. **a**–**b**, tension/compression and **c**–**h**, von Mises stress contour plots of the pleurodire *Emydura subglobosa* in **a**, **c**, **f**, dorsal, **b**, **d**, **g**, ventral and **e**, **h**, left lateral views. Models **a**–**e**, without and **f**–**h**, with the trochlea explicitly simulated. **a**–**b**, tension/compression and **c**–**h**, von Mises stress contour plots of the pleurodire *Chelodina reimanni* in **a**, **c**, **f**, dorsal, **b**, **d**, **g**, ventral and **e**, **h**, left lateral views. Models **a**–**e**, without and **f**–**h**, with the trochlea explicitly simulated.

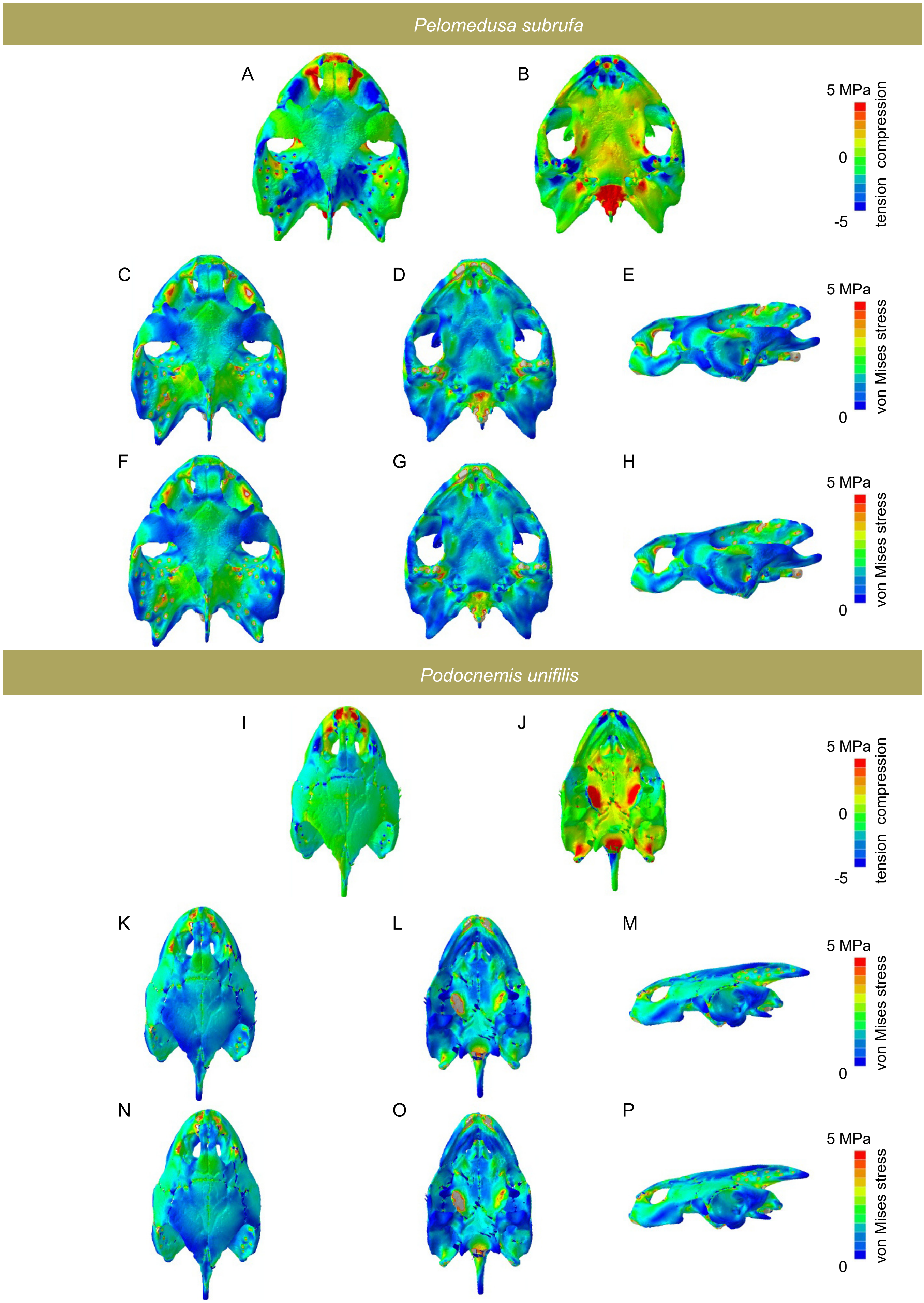

Supplementary Figure 4. **a–b**, tension/compression and **c–h**, von Mises stress contour plots of the pleurodire *Pelomedusa subrufa* in **a**, **c**, **f**, dorsal, **b**, **d**, **g**, ventral and **e**, **h**, left lateral views. Models **a–e**, without and **f–h**, with the trochlea explicitly simulated. **a–b**, tension/compression and **c–h**, von Mises stress contour plots of the pleurodire *Podocnemis unifilis* in **a**, **c**, **f**, dorsal, **b**, **d**, **g**, ventral and **e**, **h**, left lateral views. Models **a–e**, without and **f–h**, with the trochlea explicitly simulated.

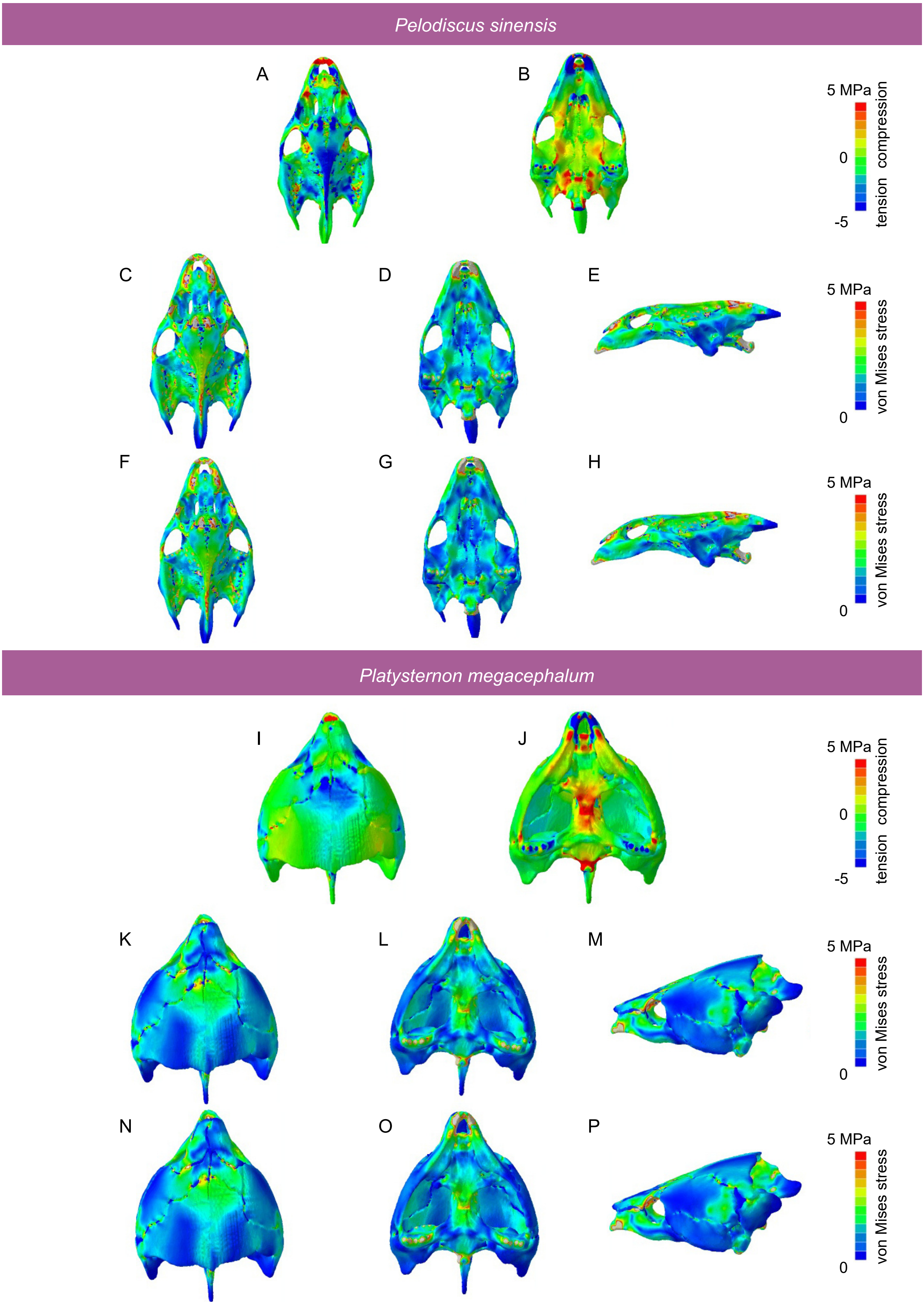

Supplementary Figure 5. **a–b**, tension/compression and **c–h**, von Mises stress contour plots of the pleurodire *Pelodiscus sinensis* in **a**, **c**, **f**, dorsal, **b**, **d**, **g**, ventral and **e**, **h**, left lateral views. Models **a–e**, without and **f–h**, with the trochlea explicitly simulated. **a–b**, tension/compression and **c–h**, von Mises stress contour plots of the pleurodire *Platysternon megacephalum* in **a**, **c**, **f**, dorsal, **b**, **d**, **g**, ventral and **e**, **h**, left lateral views. Models **a–e**, without and **f–h**, with the trochlea explicitly simulated.

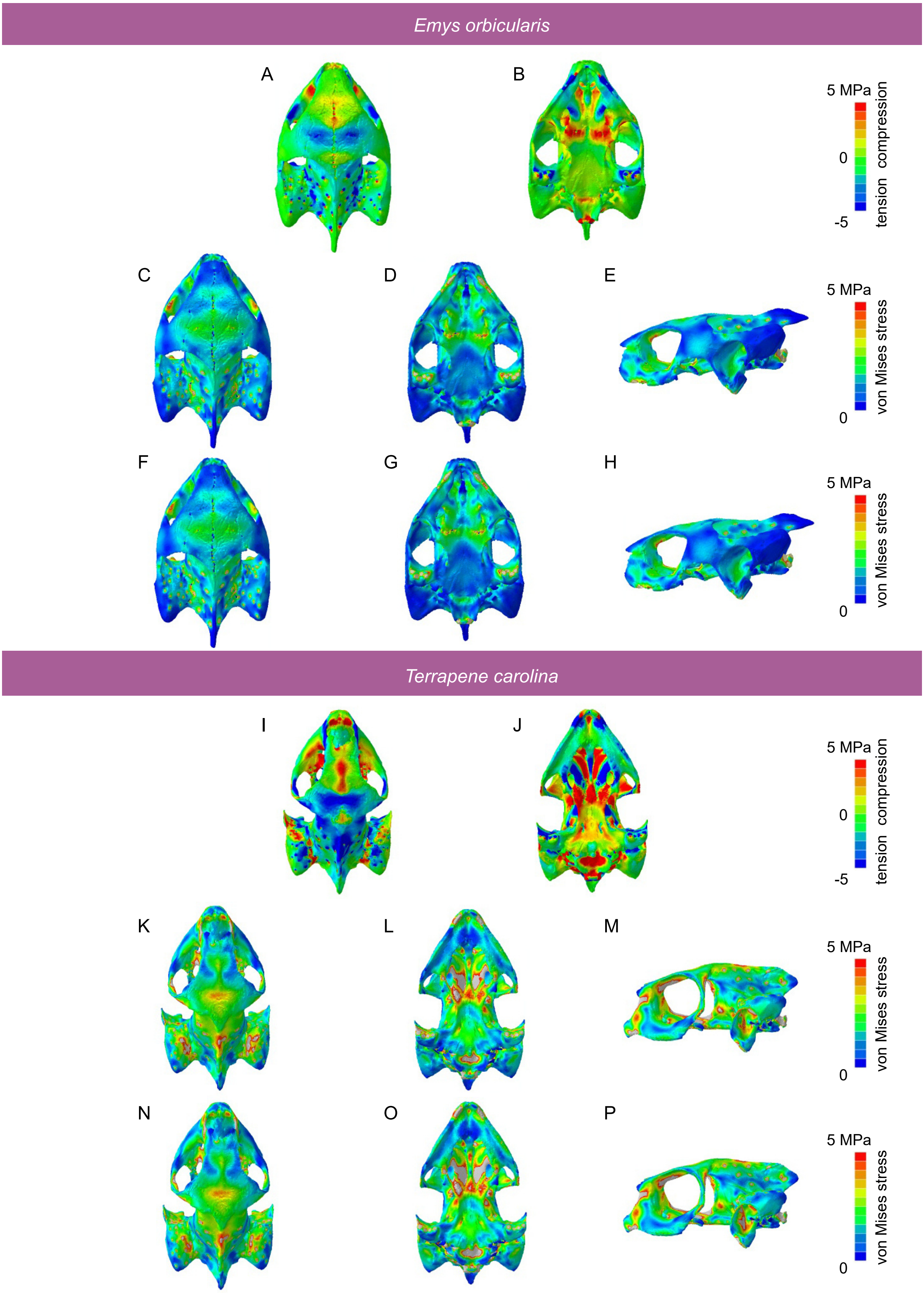

Supplementary Figure 6. a–b, tension/compression and **c–h**, von Mises stress contour plots of the pleurodire *Emys orbicularis* in **a**, **c**, **f**, dorsal, **b**, **d**, **g**, ventral and **e**, **h**, left lateral views. Models **a–e**, without and **f–h**, with the trochlea explicitly simulated. **a–b**, tension/compression and **c–h**, von Mises stress contour plots of the pleurodire *Terrapene carolina* in **a**, **c**, **f**, dorsal, **b**, **d**, **g**, ventral and **e**, **h**, left lateral views. Models **a–e**, without and **f–h**, with the trochlea explicitly simulated.

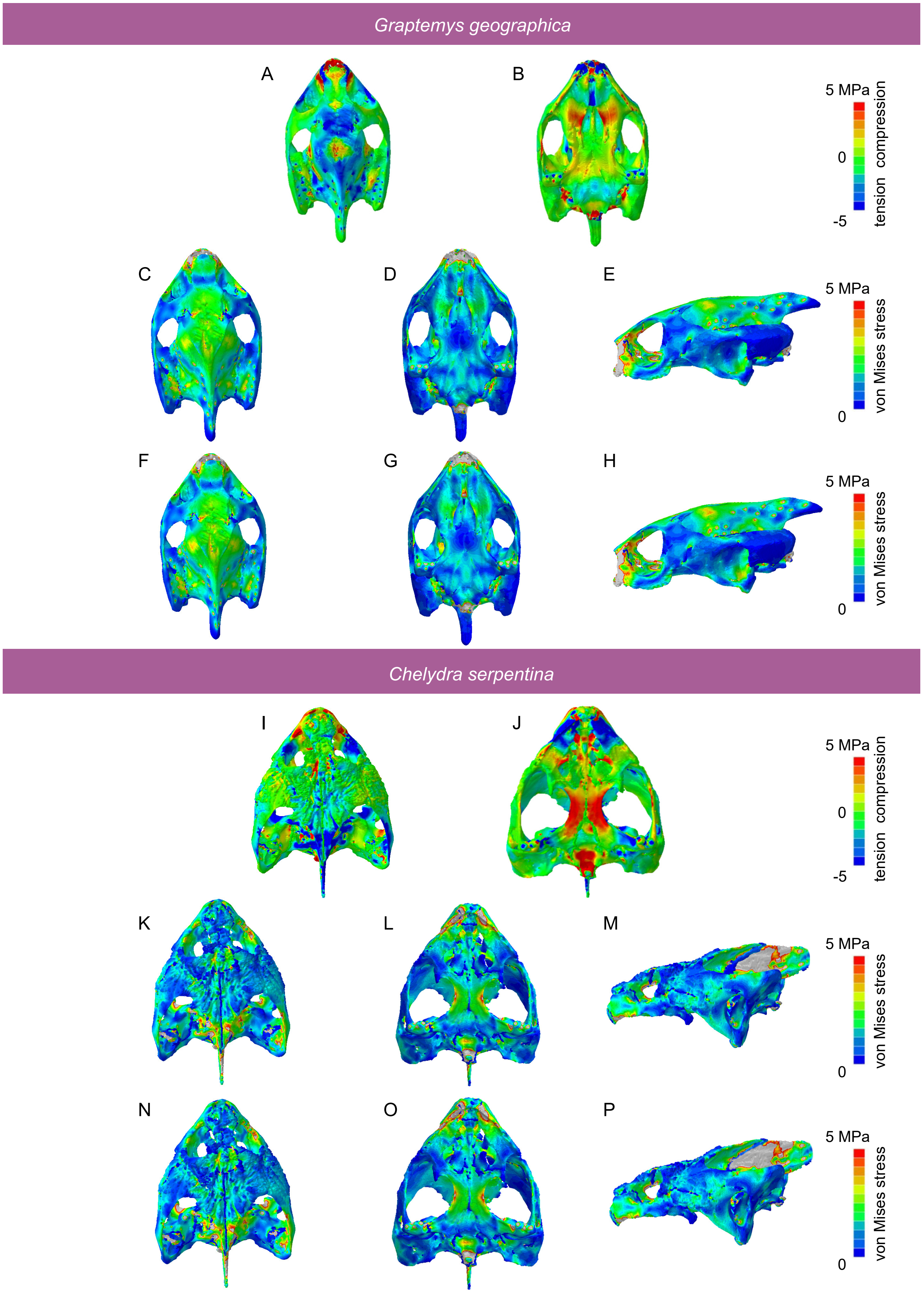

Supplementary Figure 7. a–b, tension/compression and **c–h**, von Mises stress contour plots of the pleurodire *Graptemys geographica* in **a**, **c**, **f**, dorsal, **b**, **d**, **g**, ventral and **e**, **h**, left lateral views. Models **a–e**, without and **f–h**, with the trochlea explicitly simulated. **a–b**, tension/compression and **c–h**, von Mises stress contour plots of the pleurodire *Chelydra serpentina* in **a**, **c**, **f**, dorsal, **b**, **d**, **g**, ventral and **e**, **h**, left lateral views. Models **a–e**, without and **f–h**, with the trochlea explicitly simulated.

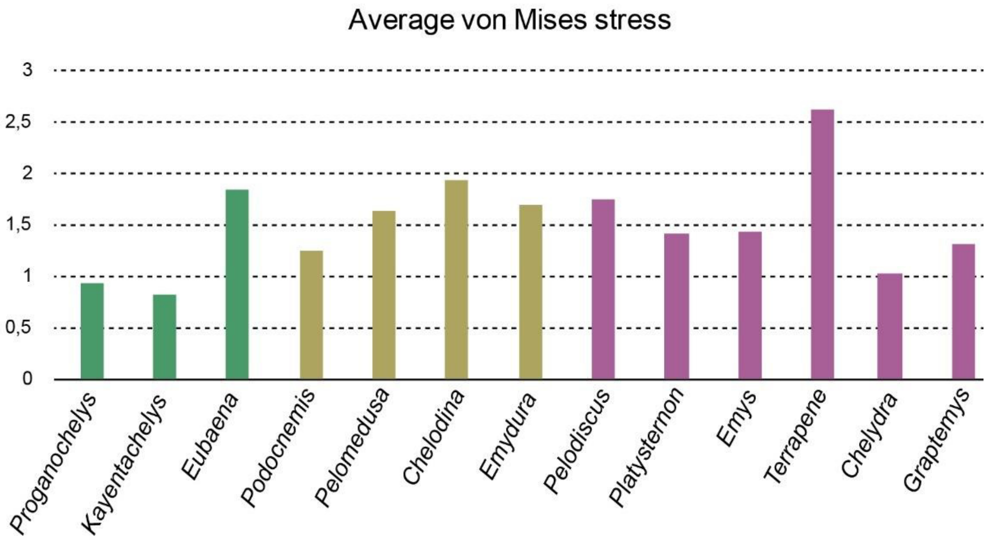

Supplementary Figure 8. Average von Mises stress for each of the analyzed taxon. Green: stem turtles, yellow: pleurodires, and purple: cryptodires.

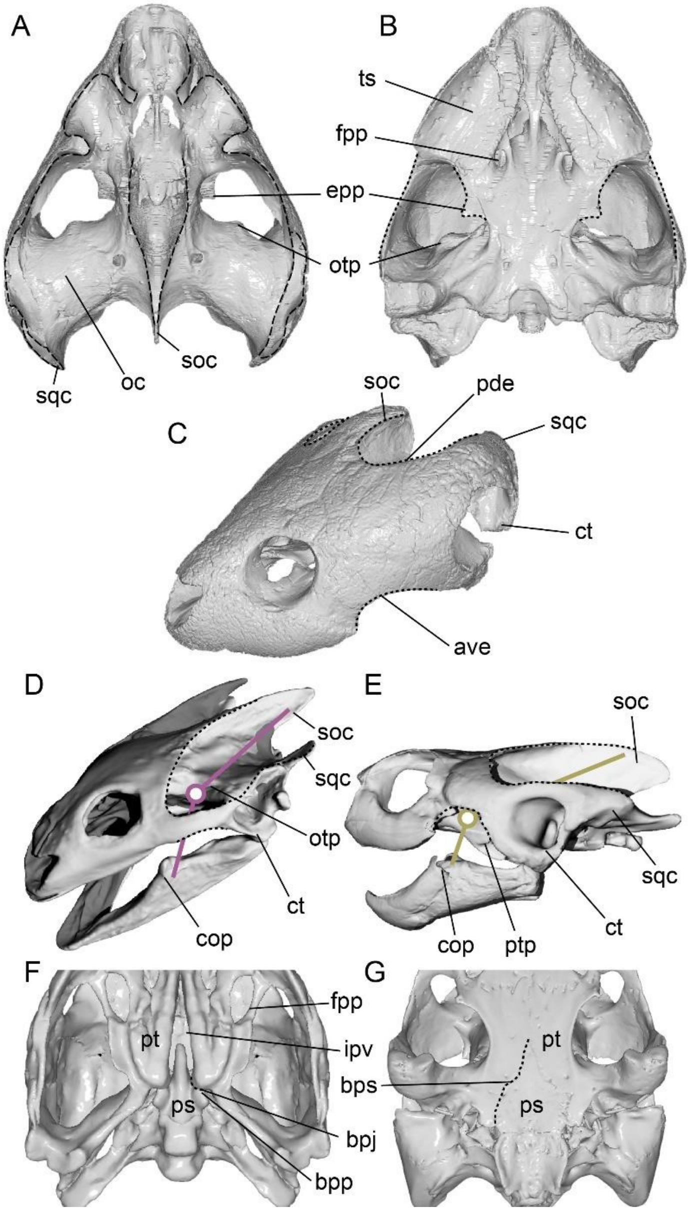

Supplementary Figure 9. Skull morphology of **a–c**, †*Eubaena cephalica*, **d**, *Pelodiscus sinensis*, **e**, *Pelomedusa subrufa*, **f**, †*Proganochelys quenstedtii*, and **g**, *Emys orbicularis* in **a**, dorsal view without temporal roof, **b, f, g**, ventral view and **c–e**, left lateral view. **d–e**, main course of external adductor muscles and trochlea location are plotted in purple (cryptodiran) and yellow (pleurodiran) lines and circles, respectively. Dotted curves represent the edges of the emarginations. Abbreviations: ave, anteroventral emargination; bpj, basipterygoid joint; bpp, basipterygoid process; bps, basipterygoid suture; cop, coronoid process; ct, tympanic cavity; epp, external process of the pterygoid; fpp, foramen palatinum posterius; ipv, interpterygoid vacuity; oc, otic chamber; otp, otic trochlear process; pde, posterodorsal emargination; pt, pterygoid bone; ptp, pterygoid trochlear process; ps, parabasisphenoid; soc, supraoccipital crest; sqc, squamosal crest; ts, triturating surface.

Supplementary Table 1. Estimated muscle and bite forces from the FE models and calculated bite efficiency (muscle force divided by bite force).

	**Standard simulation**			**Trochlear simulation**		
	Muscle force [N]	Bite force [N]	Bite efficiency [%]	Muscle force [N]	Bite force [N]	Bite efficiency [%]
Proganochelys	426	74	17.37	426	0	-
Kayentachelys	676	147	21.74	676	0	-
Eubaena	1004	277	27.58	1134	274	24.16
Chelodina	838	76	9.06	947	64	6.76
Emydura	848	134	15.80	939	126	13.42
Pelomedusa	1032	208	20.15	1102	194	17.60
Podocnemis	580	174	30.30	654	167	25.55
Emys	808	136	16.83	876	126	14.39
Chelydra	665	183	27.51	732	192	26.22
Graptemys	622	174	27.96	734	189	25.74
Pelodiscus	548	137	25.25	602	125	20.76
Platysternon	1098	280	25.50	1210	164	13.55
Terrapene	732	154	21.03	842	144	17.11

Supplementary Table 2. Summary of statistical tests of estimated bite efficiency from our FEAs and empirical bite force measures scaled to carapace size from Herrel et al.[33]. ANOVA and Pairwise comparison (Tukey HSD) tests were conducted using functions *aov* and *TukeyHSD* implemented in R environment.

FEA estimated bite efficiency

ANOVA	**Df**	**Sum Sq**	**Mean Sq**	**F value**	**Pr (>|t|)**
	2	64.7	32.37	0.846	0.458
**Residuals**	10	382.6	38.26		

Pairwise comparison

	**Estimate**	**Std. Error**	**t value**	**Pr (>|t|)**
**Pleurodira - Cryptodira == 0**	-5.186	3.993	-1.299	0.426
**Stem - Cryptodira == 0**	-1.78	4.374	-0.407	0.913
**Stem - Pleurodira == 0**	3.406	4.724	0.721	0.756

Empirical scaled bite force

ANOVA

	**Df**	**Sum Sq**	**Mean Sq**	**F value**	**p-value**
**taxon**	1	0.193	0.1932	0.959	0.336
**Residuals**	26	5.239	0.2015		

Pairwise comparison

	**Estimate**	**Std. Error**	**t value**	**Pr (>|t|)**
**Pleurodira - Cryptodira == 0**	-0.2025	0.2067	-0.979	0.336

Supplementary Table 3. Computed tomography parameters and further information for each of the sampled dry specimens used for the building of FE models.

Taxon	Classification	Collection Number	Scanning Facility	Scanner	Exposure time (s)	Voltage (kV)	Current (µA)	Projections	Voxel size (µm)
Proganochelys quenstedti	Stem turtle	SMNS 16980	Riedberg Campus of Goethe Uni. Frankfurt, Germany	Phoenix Nanotom m					25
Kayentachelys aprix	Stem turtle	TMM 43670-2	University of Texas High-Resolution X-Ray CT Facility	NSI	1	120	165	3000	31
Eubaena cephalica	Stem turtle	DMNH 96004	Uni. Fribourg, Switzerland	Bruker Skyscan 2211	0.25	180	78	1800	37
Pelodiscus sinensis	Cryptodira	IW 576-2	Steinmann-Institut für Geologie, Mineralogie und Paläontologie	Phoenix v | tome | x s 180/240					56
Platysternon megacephalum	Cryptodira	SMF 58702	Universität Tübingen	Medical Scanner					300
Emys orbicularis	Cryptodira	WGJ 1987a	Steinmann-Institut für Geologie, Mineralogie und Paläontologie	Phoenix v | tome | x s 180/240					33
Chelydra serpentina	Cryptodira	UFR VP1	Uni. Fribourg, Switzerland	Bruker Skyscan 2211	0.08	80	550	1800	35
Graptemys geographica	Cryptodira	NHMUK 55.12.6.11	Natural History Museum, London: Imaging and Analysis Centre	Nikon Metrology HMX ST 225	0.5	180	190	3142	34.53
Terrapene ornata	Cryptodira	FMNH 23014	University of Chicago PaleoCT Facility (Luo Lab)	Phoenix v | tome | x s 240	0.5	80	200	1600	29
Chelodina reimanni	Pleurodira	ZMB Herp 49659	Museum für Naturkunde Berlin	Phoenix Nanotom Xray|s				1000	28
Emydura subglobosa	Pleurodira	PIMUZ lab# 2009.37	Anthropologisches Institut der Universität Zürich	Scanco® micro-CT 80					36
Pelomedusa subrufa	Pleurodira	IW 938-1	Steinmann-Institut für Geologie, Mineralogie und Paläontologie	Phoenix v | tome | x s 180/240					48
Podocnemis unifilis	Pleurodira	SMF 55470	Steinmann-Institut für Geologie, Mineralogie und Paläontologie	Phoenix v | tome | x s 180/240					

Supplementary Table 4. Computed tomography parameters and further information for each of the PTA-stained specimens used for sampling muscle attachment sites and cross-sectional areas.

Taxon	Classification	Collection Number	Scanning Facility	Scanner	Exposure time (s)	Voltage (kV)	Current (µA)	Projections	Voxel size (µm)
Pelodiscus sinensis	Cryptodira	IW w/o number	Museum für Naturkunde Berlin	Phoenix Nanotom Xray|s	0.1	100	175	1440	14
Platysternon megacephalum	Cryptodira	R 12559	µ-CT Facility Inst. Paläontologie of the Uni. Wien, Austria	SkyScan 1173	0.12	130	61	1200	28
Chelydra serpentina	Cryptodira	R 14442	µ-CT Facility Inst. Paläontologie of the Uni. Wien, Austria	SkyScan 1173	0.12	130	61	1200	28
Graptemys pseudogeographica	Cryptodira	SMNS 3702	µ-CT Facility Inst. Paläontologie of the Uni. Wien, Austria	SkyScan 1173	0.12	130	61	1200	28
Emys orbicularis	Cryptodira	SMNS 11390	µ-CT Facility Inst. Paläontologie of the Uni. Wien, Austria	SkyScan 1173	0.12	130	61	1200	11
Terrapene carolina	Cryptodira	SMNS 7434	µ-CT Facility Inst. Paläontologie of the Uni. Wien, Austria	SkyScan 1173	0.12	130	61	1200	16
Chelodina sp.	Pleurodira	IW 1839	Museum für Naturkunde Berlin	Phoenix Nanotom Xray|s	0.1	90	200	1440	14
Emydura subglobosa	Pleurodira	IW 29	µ-CT Facility Inst. Paläontologie of the Uni. Wien, Austria	SkyScan 1173	0.12	130	61	1200	11
Pelusios niger	Pleurodira	SMNS 4625	µ-CT Facility Inst. Paläontologie of the Uni. Wien, Austria	SkyScan 1173	0.12	130	61	1200	16
Podocnemis erythrocephala	Pleurodira	SMNS 6063	µ-CT Facility Inst. Paläontologie of the Uni. Wien, Austria	SkyScan 1173	0.12	130	61	1200	13

Institutional abbreviations. *DMNH*, Denver Museum of Nature and Science, Denver, USA; *FMNH*, Field Museum of Natural History, Chicago, USA; *IW*, Ingmar Werneburg Private Collection, Tübingen, Germany; *PIMUZ*, Laboratory collection of Paläontologisches Institut und Museum der Universität Zürich, Switzerland; *SMF*, Senckenberg Museum Frankfurt, Germany; *SMNS*, Staatliches Museum für Naturkunde Stuttgart, Germany; *TMM*, Texas Memorial Museum, Austin, USA; *WGJ*, Walter G. Joyce Private Collection, Fribourg, Switzerland; *ZMB*, Zoologisches Museum Berlin, Germany.
